# Signaling pathway cross talk in Alzheimer’s disease

**DOI:** 10.1186/1478-811X-12-23

**Published:** 2014-03-28

**Authors:** Juan A Godoy, Juvenal A Rios, Juan M Zolezzi, Nady Braidy, Nibaldo C Inestrosa

**Affiliations:** 1 Centro de Envejecimiento y Regeneración (CARE); Departamento de Biología Celular y Molecular; Facultad de Ciencias Biológicas, Pontificia Universidad Católica de Chile, Av. Alameda 340, Santiago, Chile; 2 Departamento de Biología, Facultad de Ciencias, Universidad de Tarapacá, Arica, Chile; 3 Center for Healthy Brain Ageing, School of Psychiatry, Faculty of Medicine, University of New South Wales, Sydney, Australia

**Keywords:** Neurodegeneration, Cognitive decline, Neuronal network failure, Reactive oxygen species, Alzheimer’s disease

## Abstract

Numerous studies suggest energy failure and accumulative intracellular waste play a causal role in the pathogenesis of several neurodegenerative disorders and Alzheimer’s disease (AD) in particular. AD is characterized by extracellular amyloid deposits, intracellular neurofibrillary tangles, cholinergic deficits, synaptic loss, inflammation and extensive oxidative stress. These pathobiological changes are accompanied by significant behavioral, motor, and cognitive impairment leading to accelerated mortality. Currently, the potential role of several metabolic pathways associated with AD, including *Wnt* signaling, 5' adenosine monophosphate-activated protein kinase (AMPK), mammalian target of rapamycin (mTOR), Sirtuin 1 (Sirt1, silent mating-type information regulator 2 homolog 1), and peroxisome proliferator-activated receptor gamma co-activator 1-α (PGC-1α) have widened, with recent discoveries that they are able to modulate several pathological events in AD. These include reduction of amyloid-β aggregation and inflammation, regulation of mitochondrial dynamics, and increased availability of neuronal energy. This review aims to highlight the involvement of these new set of signaling pathways, which we have collectively termed “anti-ageing pathways”, for their potentiality in multi-target therapies against AD where cellular metabolic processes are severely impaired.

## Lay abstract

Alzheimer's disease (AD) is characterized by the progressive loss of cholinergic neurons leading to dementia. Deciphering the molecular basis underlying this multifactorial neurodegenerative disorder remains a significant challenge. Increased oxidative stress and misfolded protein formations are the basis of AD. Recently, the several new cellular signaling pathways have been implicated in the pathobiology of AD. These include *Wnt* signaling, 5' adenosine monophosphate-activated protein kinase (AMPK), mammalian target of rapamycin (mTOR), Sirtuin 1 (Sirt1, silent mating-type information regulator 2 homolog 1), and peroxisome proliferator-activated receptor gamma co-activator 1-α (PGC-1α). These new signaling pathways may provide new therapeutic targets to slow down or prevent the development of AD.

## Introduction

**Figure 1:**
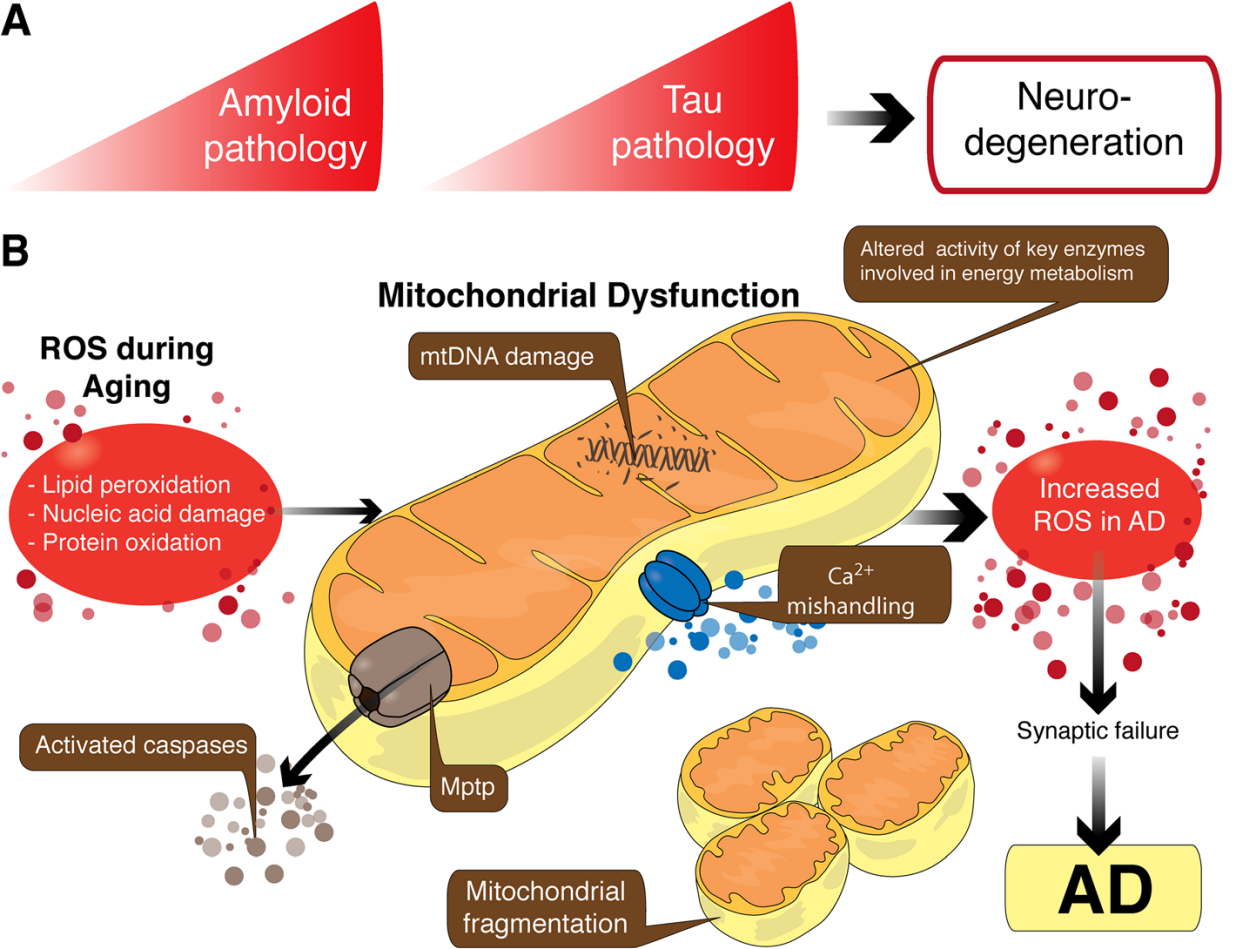
**Hallmarks of AD, progression of the disease and mitochondrial dysfunction. A**: The diagram shows the hallmarks in AD. **B**: The multiple pathogenic mechanisms contributing to the pathological hallmarks of AD consist of increased of ROS production, Aβ-induced mitochondrial dysfunction, and apoptosis due to impairment of mitochondrial Ca^2+^ handling ability, altered Ca^2+^ homeostasis, increased mitochondrial permeability transition pore opening, and promotion of cytochrome *c* release. Aβ inhibits protein import inside the mitochondria. APP also alters Ca^2+^ homeostasis leading to apoptosis. Mitochondrial DNA mutations and mitochondrial DNA damage are also involved in the pathogenesis of AD, and are associated with synaptic and neuronal loss, amyloid plaques, and NFTs. Perturbed cerebral energy metabolism plays a central role in multiple pathogenic cascades of AD. Abbreviations: AD, Alzheimer’s disease; Ca^2+^, calcium; Mptp, mitochondrial permeability transition pore; ROS, reactive oxygen species.

Alzheimer’s disease (AD) is a debilitating neurodegenerative disorder characterized by the progressive loss of cholinergic neurons, leading to the onset of severe behavioral, motor and cognitive impairments. In order to establish the criteria that would accurately define AD, patients with senile dementia were traditionally excluded since, despite its similarity, senile dementia was generally considered an age-associated phenomenon, and not a true disease. However, since extracellular amyloid β (Aβ) plaques and intracellular neurofibrillary tangles (NFTs) containing hyper-phosphorylated tau, are frequently present in the brain of patients with senile dementia, investigators eventually expanded the definition of AD to also include those with senile dementia, plaques and tangles (Figure 1) [1].

Energy demands and calcium fluctuation within neuronal synapses are the prerequisite of neuronal communication; to meet this process, the mitochondria are enriched in synapses for site-directed energy provision and calcium homeostasis. Reduced energy metabolism, particularly related to low levels of glucose, in the diseased brain is one of the best documented metabolic abnormalities in AD. In fact, the decline in baseline glucose metabolism is viewed as a sensitive measure, useful for monitoring change in cognition and functionality in AD. Deficits in mitochondrial function and increased Aβ accumulation in synapses lead to reduced synaptic activity and consequent neuronal damage. Such synaptic alteration and mitochondrial dysfunction have been observed in many neurodegenerative disorders including AD. The normal physiological function of the mitochondria is dependent upon their intact structure to maintain the electrochemical gradient. Structurally damaged mitochondria, as evidenced by partial or near complete loss of the internal structure and cristae, are abundant and represent a prominent feature in dystrophic neurons in postmortem AD brains [2]. On the other hand, calcium (Ca^2+^) mishandling has been reported in peripheral cells isolated from AD patients, with the endoplasmic reticulum (ER) developing calcium overload due to reduced calcium uptake (Figure 1) [3].

Mitochondria are highly metabolic organelles that combine nutrient sensing and growth signaling pathways to regulate health span and longevity by maintaining energy production and Ca^2+^ homeostasis, and reducing apoptosis. Genetic approaches have identified several signaling pathways which represent critical modifiers of mitochondrial function. These pathways have been shown to increase the transcription of important mitochondrial genes following exposure to oxidative and inflammatory insult within the cell. Among these signaling pathways are the *Wnt* signal transduction pathways, 5' adenosine monophosphate-activated protein kinase (AMPK), mechanistic target of rapamycin (mTOR) complexes, and activation of the Sirtuin 1 (silent mating-type information regulator 2 homolog 1)/peroxisome proliferator-activated receptor gamma co-activator 1-α (Sirt1/PGC-1α) axis.

In this review, we will focus on already published evidence that allows, based in our own experience, to propose a potential connection between several mechanisms already described as neurodegenerative/AD-related and how these signaling pathways will contribute to AD. We consider that a greater understanding of the molecular basis of these pathways and how they interact within the cell will foster efforts to slow down or attenuate metabolic deficits that are observed in AD.

### Role of *Wn*t signaling in neuronal synaptogenesis and AD

The *Wnt* signaling pathway is involved in several key cellular processes associated with cellular proliferation, differentiation, adhesion, survival, and apoptosis in several catabolic and anabolic cells, including neurons and glial cells which are the key resident cells of the Central Nervous System (CNS) [4,5]. *Wnt* proteins are a family of secreted cysteine-rich glycosylated protein that are named after the *Drosophila* protein “wingless” and the mouse protein “Int-1” [4]. Currently, 19 of the 24 *Wnt* genes expressing *Wnt* protein have been identified in humans, while 80 *Wnt* target genes have been indentified from genetic studies in human, mice, *Drosophila*, *Xenopus*, and Zebrafish populations [4,5]. *Wnt* binds to Frizzled (Fz) transmembrane receptors located on the cell surface leading to the induction of at least three distinct downstream signaling pathways [5]. The first is known as the canonical *Wnt* pathway which regulates gene transcription through β-catenin, also called *Wnt/β-catenin.* The second, is the non-canonical pathway modulates by intracellular Ca^2+^ release, also called *Wnt/Ca*^*2+*^, and the third one, the *Wnt* cell polarity, in which the Jun N-terminal kinase (JNK) plays a role, also called the *Wnt/PCP-JNK* pathway [6-8].

#### Wnt Signaling protects synaptic integrity from Aβ toxicity

Numerous studies have shown that *Wnt* signaling components are altered in AD: (a) among the *Wnt* components that are affected in AD, it was shown that β-catenin levels are reduced in AD patients carrying presenilin-1 (PS1)-inherited mutations [9]; (b) exposure of cultured hippocampal neurons to Aβ results in the inhibition of canonical *Wnt* signaling [10,11]; (c) Dickkoff-1 (Dkk1) a *Wnt* antagonist is induced by the Aβ protein in hippocampal neurons [12]; and it is elevated in post-mortem brain samples from AD patients and brains from transgenic AD animal models [13,14]; (d) Dkk3, highly related to Dkk1, is elevated in plasma and cerebrum spinal fluid from AD patients [15]; (e) apo-lipoprotein E (apoEϵ4), a risk AD factor, inhibits canonical *Wnt* signaling [16]; (f) a common genetic variation within the low-density lipoprotein receptor-related protein 6 (LRP6) lead to disease progression [17]; (g) Dkk1 reversibly reduces the amount of synaptic proteins and the number of active pre-synaptic sites, inducing synaptic disassembly at pre- and postsynaptic sites [18,19]; (h) clustering, a susceptibility factor for late-onset AD, regulates Aβ amyloid toxicity via Dkk1*-*driven induction of the non-canonical *Wnt/PCP-JNK* pathway, which contributes to tau phosphorylation and cognitive impairment [20].

Synaptic failure is an early event in AD, and soluble Aβ oligomers are proposed to be responsible for the synaptic pathology that occurs prior to plaque deposition and neuronal death [21]. The non-canonical *Wnt-5a* ligand prevents the decrease in the amplitude of excitatory postsynaptic currents induced by Aβ oligomers, indicating that this ligand prevents the synaptic damage triggered by Aβ [22]. *Wnt-5a* prevents the decrease in the PSD-95 postsynaptic clusters through the *Wnt/PCP-JNK* pathway. However, *Wnt-5a* also stimulates the trafficking of GABA_A_ and NMDA receptors to the neuronal surface [23,24], the development of dendritic spines [25] and protects neuronal mitochondria from Aβ oligomers [26], through the activation of the *Wnt/Ca*^*2+*^ pathway. More recent studies, using small *Wnt* molecules to activate both canonical and non-canonical *Wnt* signaling *in vivo,* enhances cognition in adult mice and reverses cognitive deficits and LTP in the APPswe/PS-1 transgenic model of AD [27]. These studies support the idea that alterations in the *Wnt* signaling pathway, both the canonical (*Wnt*/β-catenin) and the non-canonical (*Wnt/PCP* and Wnt/Ca^2+^) are involved in the modulation of synaptic development, as well as, in the progression of AD [28].

Finally, the activation of several signaling pathways that cross talk with the *Wnt* pathway, including the nicotinic and muscarinic ACh receptors, peroxisome proliferator-activated receptor (PPAR)α and γ, antioxidants, and anti-inflammatory pathways, support the neuroprotective potential of the *Wnt* signaling cascade in AD [29-31].

#### Cholinergic system and Wnt Signaling cross-talk: ancient and new strategy

The “cholinergic hypothesis” of AD, which was developed after disturbances were found in the metabolism of acetylcholine in postmortem AD brains [32], states that there is a loss of cholinergic neurons in the basal forebrain and that the impairment of cognitive functions and the behavioral disturbances observed in patients with AD are due, in part, to cortical deficiencies in cholinergic neurotransmission. The decrease of cholinergic neurons leads to the alteration of several proteins in the cholinergic system, such as decreased activity of acetylcholinesterase (AChE) and cholineacetyl transferase [32]. We have previously shown that a macromolecule found in the synapses interacts with Aβ to form a complex which alters the normal synaptic function in hippocampal neurons [33,34]. Additionally, our group has also demonstrated that Aβ-AChE complexes were more neurotoxic than those of Aβ alone, depending on the level of AChE [34], suggesting that AChE may plays a key role in the neurodegenerative changes observed in the AD brain. Interestingly, hyperforin, a phytochemical drug which modulates acetylcholine release in the CNS, [34], is able to prevent the Aβ-induced spatial memory impairments and Aβ neurotoxicity *in vivo*[35,36]. Moreover, tetrahydrohyperforin (THH), a semi-synthetic derivative of hyperforin, restores brain AChE activity, reduces the levels of cholinergic markers associated with amyloid plaques, oxidative stress, and apoptosis, and protects cholinergic neurons in a double transgenic mouse model of AD [36,37].

A recent study has shown that a *Wnt* signaling pathway may be involved in maintaining synaptic strength in the CNS by modulating the translocation of a subset of acetylcholine receptors (AChRs) to synapses [38]. In *Caenorhabditis elegans*, mutations in the *Wnt* ligand, CWN-2, the Fz receptor, LIN-17, the Ror receptor tyrosine kinase, CAM-1, and the DSH cytoplasmic phosphoprotein, DSH-1 (involved in both canonical and non-canonical *Wnt* signaling) lead to synaptic accumulation of the AChR, a mutant α_7-_nACh receptor (ACR-16/α7), impaired synaptic function, and trigger significant behavior deficits [37,38]. Results of this study suggest that synaptic plasticity is mediated, at least partly, by *Wnt* signaling.

#### Reducing oxidative stress by Wnt signaling activation

Aβ accumulation is believed to plays a key role in the cognitive deficits observed in AD patients. There is evidence relating the etiopathology of the disease with free radicals [39]. Through *in vitro* experiments it has been shown that one of the neurotoxicity mechanism of Aβ peptides is through oxidative stress, and inhibitors of catalase-Aβ interactions protect from Aβ toxicity [40]. Moreover, the enhancement of the oxidative state by the *in vivo* depletion of vitamin E has been shown to result in an increased amount of Aβ by the inhibition of it clearance from the brain [41]. Previously, we have shown that the peroxisomal proliferation, simultaneously with an increase in catalase, is able to protects against the neurotoxicity of Aβ in cultured rat hippocampal neurons, leading to significant improvements in spatial memory, lower levels of Aβ aggregates, reduced glial activation, decreased tau phosphorylation, and increased postsynaptic proteins and long-term potentiation (LTP) [42].

*Wnt* signaling may also confer neuroprotection against oxidative stress in AD. *Wnt1* overexpression has been shown to protect neurons against Aβ-mediated oxidative stress, and oxidative DNA damage in primary hippocampal murine neurons [4]. Reduced *Wnt* activity may also increase the vulnerability of neuronal cells to oxidative insult [43]. In AD, Aβ toxicity can induce the expression of glycogen synthase kinase 3 (GSK-3β), a serine/threonine protein kinase which phosphorylates β-catenin, and thus leading to its depletion [5,18]. Also, reduced production of Aβ can occur in response to increased PKC activity which is regulated by the *Wnt* pathway [5,18]. Overexpression of DSH-1 and DSH-2 has been shown to inhibit GSK-3β mediated phosphorylation of tau protein, thus preventing the formation of NFTs (one of the main pathological hallmarks of AD), and increasing neuroprotection [44].

### Cross-talk between AMPK and mTOR pathway

**Figure 2:**
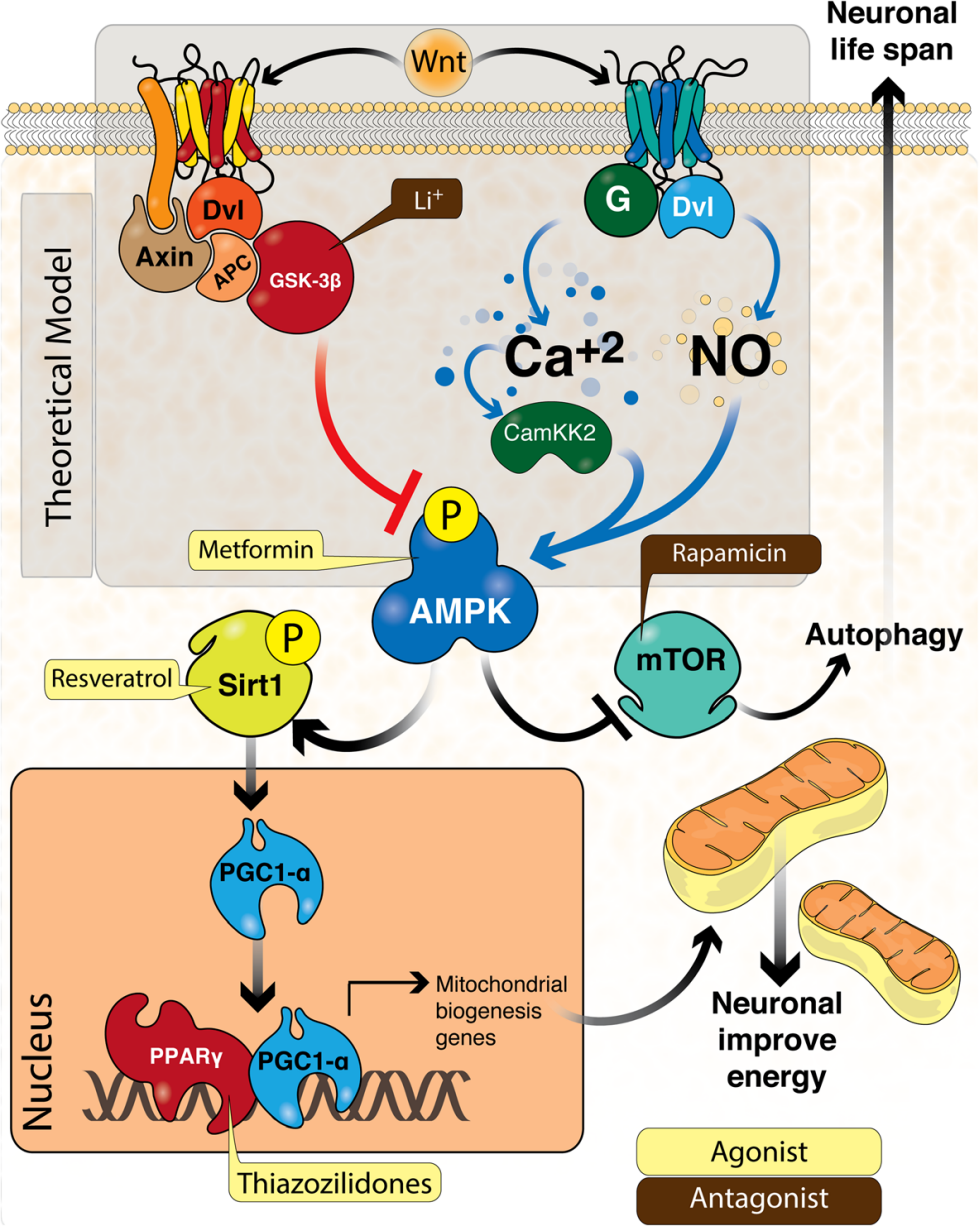
**Interplay between *Wnt *signaling and other anti-ageing pathways.** In this scheme we show the integration and interaction of multiple signaling pathways: the first line (top) shows a *Wnt* ligand, binding Frizzled receptor and activated canonical and non-canonical *Wnt* signaling. The canonical pathway (left) leads to GSK3β inhibition. AMPK activation is known to inhibit by GSK3β. The non-canonical pathway (right) increases intracellular Ca^2+^ levels. Nitric oxide (NO), a second messenger, is known to directly activate AMPK. AMPK is also activated by Ca^2+^ through CaMKK2. Therefore, AMPK activation by the Wnt pathway represents a hypothetical concept (“theoretical model” in transparent gray box). In the second line, AMPK leads to activation of Sirt1 (right). Sirt1 de-acetylates PGC-1α, and this transcription factor translocates to the nucleus and interacts with PPARγ heterodimerization to enhance the expression of mitochondrial biogenesis genes. As well, AMPK inhibits mTOR complex (right) resulting in autophagy stimulation. Additionally, we show the established target of several compounds (Li^+^, Metformin, Rapamicin, Resveratrol and Thiazolidinediones) on these intricate inter-linking signaling pathways to neuronal energy availability and cellular life span. Abbreviations: G, G-protein-coupled receptor; Dvl, Segment polarity protein disheveled homolog DVL-1; APC, adenomatous polyposis coli protein; Ca^2+^, calcium; Ca^2+^/CaM-dependent protein kinase kinase β (CaMKKβ); AMPK, 5' adenosine monophosphate-activated protein kinase; mTOR, mechanistic target of rapamycin, Sirt1, silent mating-type information regulator 2 homolog 1; PGC-1α, peroxisome proliferator-activated receptor gamma co-activator 1-α; GSK-3β, Glycogen synthase kinase 3; P,PPARα, phosphorylation; peroxisome proliferator-activated receptor alpha; Li^+^; lithium.

AMPK is a heterotrimeric protein kinase complex expressed widely in most cell and tissue types. The primary function of AMPK is to act as a sensor of intracellular ATP levels and is coupled to phosphorylation of downstream substrates of ATP producing pathways [45]. The regulation of AMPK involves two upstream pivotal enzymes: Serine/threonine kinase 11 (STK11) also known as liver kinase B1 (LKB1), the Ca^2+^/CaM-dependent protein kinase kinase β (CaMKKβ) and also other stimulus like as nitric oxide (NO) [46]. AMPK is returned to its inactive form by dephosphorylation mediated by specific phosphatases (PPase) [47]. AMPK possesses several downstream targets including enzymes associated with glycolytic pathways and lipolysis, and even "master energy regulators" [48], such as the PPARγ coactivator-1α (PGC-1α), which triggers mitochondrial biogenesis via Sirt1-mediated de-acetylation in response to AMPK activation (Figure 2) [49]. AMPK also directly phosphorylates several sites of the transcription factor, Forkhead box O3 (FOXO3), activating transcription of several genes, including some associated with resistance to oxidative stress [50]. Additionally, AMPK inhibits protein synthesis by direct phosphorylation of Raptor and ULK1, a novel serine/threonine kinase and subunit of the mTORC1 complex, triggering autophagy to recycle amino acids and other cell components during cellular starvation (Figure 2) [51,52]. In neuronal cells, increased mTOR activity results in several stimuli, including BDNF, leptin and Ca^2+^ influx, and contributes to the maintenance of synaptic plasticity through regulation of protein synthesis required for the late-phase of long-term potentiation (LTP) [53]. Therefore AMPK functions as a "master of master cell physiology", and pharmacological modulation represents an attractive therapeutic target for many age-related disorders, such as neurodegenerative diseases and AD in particular.

#### Deciphering the role of AMPK-mTOR in AD

Currently the role of AMPK and mTOR in the development and progression of AD is poorly understood, and recent studies have provided evidence that AMPK and mTOR are main targets for deregulations in AD [54,55]. Controversially, *in vitro* models have shown that AMPK activation inhibits *tau* phosphorylation in rat cortical neurons [56], while others confirmed that AMPK could phosphorylate *tau* at several sites (including Thr231 and Ser396/404), and interrupts the binding of *tau* to microtubules [57,58]. On the other hand, several studies have shown that AMPK activation represses amyloidogenesis in neurons [59]. Additionally, AMPK activation decreases mTOR signaling and enhances autophagy and lysosomal degradation of Aβ [60-63]. Nevertheless, a recent study demonstrated that metformin, an oral antidiabetic drug in the biguanide class, can lead to activation of the AMPK and transcriptional up-regulation of β-secretase (BACE1), the rate-limiting enzyme for Aβ generation, at therapeutic doses, and significantly increasing the generation of both intracellular and extracellular Aβ species [64]. These findings suggest a potentially harmful effect for the use of metformin in diabetic elderly demented patients.

#### Can new and old drugs that activate AMPK prevent AD?

Several animal studies have highlighted the “anti-AD” effects of naturally occurring phytochemicals which have been shown to activate AMPK. For example, phytic acid [64], which is found in food grains could attenuate levels of ROS and Aβ oligomers in transgenic mice, and moderately up-regulate the expression of the autophagy protein (beclin-1), Sirt1 and the AMPK pathway [65]. Moreover, arctigenin, derived from *Arctium lappa*, could reduce both Aβ production by β-site amyloid precursor protein cleavage enzyme 1, and enhance Aβ clearance by potentiated autophagy by inhibition of protein kinase B PKB/mTOR signaling, and AMPK activation, and improve memory in APP/PS1 AD mice [66]. Similarly, resveratrol promotes anti-ageing pathways and previously has been described as anti-AD agent [67]. Resveratrol has been previously shown to increase cytosolic Ca^+2^ levels and enhance AMPK activation through CAMKK2 activation, promoting autophagy degradation of Aβ and reduced cerebral Aβ deposition [59]. Another study showed that curcumin could up-regulate two new regulators of tau protein, BCL2-associated athanogene 2 (BAG2), and lysosomal-associated membrane protein 1 (LAMP1) [68]. As well, methylene blue has shown neuroprotective effects in neuropathological conditions [69] by promoting macroautophagy via AMPK activation rather than inhibition of mTOR pathway *in vitro*, and robustly increased the anti-apoptotic Bcl-2 protein levels [70]. A compound named butyrolactone, a product for γ-hydroxybutyric acid (GHB), also known as 4-hydroxybutanoic acid, a naturally occurring substance found in the CNS, as well as in wine, beef, and citrus fruits, increases the levels of the insulin-degrading enzyme (IDE), suppresses autophagy via the mTOR pathway, lowers Aβ levels and prevents AD-like cognitive deficits in APP/PS1 mice [71].

Other authors have reported that topiramate (TPM) and levetiracetam (LEV), two classical drugs used in the management of epilepsy, alleviated behavioral deficits and diminished senile plaques in APP/PS1 mice. The mechanism underlying these observed effects involved increased Aβ clearance and up-regulated Aβ autophagic degradation through GSK-3β deactivation and AMPK activation [72]. Another recent study, showed that carbamazepine, an anticonvulsant and mood-stabilizing drug used primarily in the treatment of epilepsy and bipolar depression, demonstrates anti-AD effect in APP/PS1 transgenic mice via mTOR-dependent pathway and increased autophagy, leading to reduced amyloid plaque load and Aβ_42_ levels [73]. During a phase-II study, latrepirdine, an antihistaminic drug, also showed potent anti-AD effects. *In vitro*, latrepirdine stimulated mTOR and ATG5 dependent autophagy, leading to the reduction of intracellular levels of APP metabolites, including Aβ and the abrogation of behavioral deficit and autophagic malfunction in TgCRND8 mice [74]. Finally, rapamycin, which is extensively used in transplantation medicine to prevent organ rejection, represents a very attractive drug in AD because it can promote neuronal survival. However is has never been considered as a potential treatment for AD due to its potent immunosuppressive effect [75]. To date, the mechanism underlying the anti-AD properties of rapamycin are still debatable. However, it has been suggested that inhibition of mTOR by rapamycin improves cognitive deficits and rescues Aβ pathology and NFTs through increased autophagy [76-78].

### The Sirt1-PGC-1α transcriptional complex

Sirtuins are a new class of histone deacetylases dependent on the coenzyme nicotinamide adenine dinucleotide (NAD^+^) as the essential substrate. Sirtuins are widely expressed through the mammalian body, but appear to be selectively localized at the subcellular level: Sirt3, 4 and 5 are primarily mitochondrial; Sirt1, 6 and 7 are mainly nuclear; while Sirt2 is the only sirtuin located in the cytosol [79]. Sirt3 regulates mitochondrial metabolism and may sense NAD^+^ levels in the mitochondria, since increased NAD^+^ triggers a regulatory pathway that would activate Sirt3 leading to the deacetylation of specific targets [80]. It has been demonstrated that mice deficient in Sirt3 present hyperacetylation [81] of the metabolic enzyme glutamate dehydrogenase (GDH), suggesting that Sirt3 may have profound impact on metabolic control [82].

Recent evidence suggests that mitochondrial biogenesis is regulated in part by PGC-1α, a transcriptional co-activator of PPARγ, as well as other transcription factors [83]. It was therefore of considerable interest when it was shown that PGC-1α activity was dependent on Sirt1-deacetylation [84]. Despite this, the role of PGC-1α in AD remains unclear. Reduced PGC-1α expression has been previously reported in brains of AD patients, and Tg2576 mice which have developed insulin resistance following chronic feeding with a high fat diet [85]. As well, PGC-1α and its closely related isoform, PGC-1β, are abundantly expressed and widely distributed in the brain, where they are thought to exhibit interchangeable roles for certain functions, such as maintenance of neuronal mitochondrial biogenesis [86].

Sirt1 has been shown to function together with PGC-1α to promote adaptation to caloric restriction by regulating the genetic programs for gluconeogenesis and glycolysis in the liver. Sirt1 interacts with and deacetylates PGC1α at multiple lysine sites, increasing PGC-1α activity and leading to the induction of liver gluconeogenic genes transcription [87]. This interaction suggests that the Sirt1-PGC-1α transcriptional complex may represent a core component of the brain neural circuitry concerned with modulating energy homeostasis.

#### *PGC-1*α: a bioenergetics sensor in AD

It has been suggested that mitochondrial biogenesis might be regulated by tissue energetic status, and that sirtuins may represent important energy sensors in this homeostatic loop. Indeed, the notion that PGC1α acetylation and function, and by extension mitochondrial activity, are regulated in a nutrient-dependent fashion by Sirt1 is appealing. Nonetheless, the concept that Sirt1 in turn functions in response to nutrient-sensitive changes in basal NAD^+^ levels, although often invoked, until recently has had little experimental support [88]. Resveratrol, a Sirt1 activator, induces mitochondrial biogenesis and protects against metabolic decline, but whether Sirt1 mediates these benefits is the subject of continuous debate. Interestingly, studies conducted in adult Sirt1 conditional-knockout mice have shown that resveratrol-mediated AMPK activation is dose-dependent, and that the Sirt1 is the key effector of this interaction. These data indicate that Sirt1 plays an essential role in stimulating AMPK, and improves mitochondrial function both *in vitro* and *in vivo*[89].

The Sirt1-PGC-1α transcriptional complex has recently been implicated in the pathogenesis of AD. One study showed that the transcription of BACE1 is modulated by up- or down regulation of PGC-1α *in vitro* and *in vivo*, in eNOS-deficient mouse brains exposed to a high fat diet [90]. Modest fasting in these mice showed reduced BACE1 transcription in the brains, parallel to elevated PGC-1α expression and activity. The inhibitory effect of PGC-1α was dependent on activation of PPARγ via Sirt1-mediated deacetylation in a ligand-independent manner [90]. The direct interference between Sirt1-PPARγ-PGC-1α and BACE1 represents a unique non-canonical mechanism of Sirt1-PGC1α in transcriptional repression in neurons in response to metabolic impairment.

### Exploring mitochondrial dysfunction in AD

For almost two decades, the “amyloid cascade hypothesis” has dominated our understanding of the aetiology and progression of AD. Briefly, this hypothesis suggested that accumulation of Aβ, a product of APP cleavage induces salient biochemical changes in the brain leading to the development of pathological and clinical changes observed in AD [91-93]. This hypothesis stems from the identification of an APP mutation in a family with autosomal dominant amyloid angiopathy, dementia, and AD-typical histology [94]. Two other genes that were subsequently found to contain mutations in autosomal dominant AD were PS1 and PS2 [95]. These protein form is important components of the γ-secretase complex, which is necessary for the processing of APP. While this hypothesis has been extrapolated to account for sporadic AD, it is important to note that sporadic AD patients do not have mutations in APP or PS genes, and the molecular basis for the accumulation of neurotoxic forms of Aβ is unknown [96]. The “mitochondrial cascade hypothesis” was proposed in 2004 to provide a greater explanation for the continuous correlation between advancing age and AD risk, and to provide a more accurate explanation of the biochemical abnormalities that have been observed in AD patients [97,98].

#### Revisiting the mitochondrial cascade hypothesis of sporadic AD

The “mitochondrial cascade hypothesis” emerged in response to the growing body of evidence for AD-related mitochondrial dysfunction [97,98]. Many studies have demonstrated that Aβ can induce mitochondrial abnormalities. Since 1983, studies using transgenic animal models have demonstrated alterations in mitochondrial enzymes in the Alzheimer’s disease brain. Moreover, FDG PET studies have shown that in AD, decreased glucose metabolism precedes clinical diagnosis. This could be interpreted as an early clinical finding of mitochondrial failure in AD [99,100].

The main evidence implicating mitochondrial dysfunction in AD can be summarized by at least five general statements: (i) reduced energy metabolism due to alterations in the key enzymes involved in oxidative phosphorylation, are associated with reduced neuronal expression of nuclear genes encoding subunits of the mitochondrial electron transport chain [101], (ii) Ca^2+^ imbalance through impaired buffering capacity and modifications to the endoplasmic reticulum (ER) Ca^2+^ channels leads to neuronal apoptosis, triggered by the calmodulin-dependent kinase and calpain activations [102,103]; (iii) abnormal mitochondrial dynamics have revealed significantly reduced mitochondrial length. In biopsied AD brains, biochemical data collectively suggest that there is likely enhanced fission, overexpression of dynamin-like protein 1 protein (DLP1) and down regulation of the optic atrophy protein 1 (OPA1) [104]; (iv) mitochondrial biogenesis is regulated by the Sirt1-PGC-1α axis and nuclear respiratory factor (NRF). In hippocampal tissues from AD patients and APP mice M17 cells, the levels of PGC-1α, NRF1and NRF2 were significantly decreased in comparison with healthy patients and wild type mice [105]. In this sense, PGC-1α overexpression has been shown to be neuroprotective both in *in vitro* and *in vivo* in several models for neurodegenerative diseases. Contrary to these findings, a recent study showed that continuous PGC-1α overexpression was cytotoxic to dopaminergic neurons *in vivo*[106,107]; (v) finally, by products of macromolecular oxidation, such as 4-hydroxynonenal (4-HNE), which is produced by lipid peroxidation in cells, may facilitate the self-assembly of *tau* protein into fibrillar polymers similar to those found in paired helical filaments (PHF), present in the brain of AD patients. These result strong suggest that oxidative stress, either by itself or as part of a “two hit process”, causes neuronal dysfunction, and AD [108].

#### Overcoming mitochondrial damage as an anti-ageing approach

Tremendous investments in basic research have been focused on preserving mitochondrial function in AD. Multiple approaches include strategies aimed at increasing mitochondrial mass, promoting fusion-fission balance, preventing mitochondrial Ca^+2^ overload, avoiding membrane swelling and improving the overall redox status. Novel therapeutics which exert a positive effect on these targets, may reinforce energy delivery from the mitochondria [109]. In this regard, dietary zeolite (micronized zeolite) supplementation has been shown to reduce mitochondrial ROS, increase superoxide dismutase (SOD) levels, and attenuate Aβ accumulation in the APP/PS1 mouse brain [110]. In the same way, *Salvia sahendica* extracts prevented reduction in the level of NRF1 and mitochondrial transcription factor A (TFAM), induced by Aβ [111]. In another study, melatonin and caffeine treatment, almost completely restored mitochondrial function in assays of respiratory rate, membrane potential, ROS production, and ATP level in the brains of the APP/PS1 [112]. Apigenin, a poor toxic and non-mutagenic subclass of flavonoid, has been reported to reduce the toxic effects of Aβ, although it did not provide a sufficient effect on decreasing APP expression and Aβ burden; However, restitution of redox balance due to increased intracellular glutathione levels and potentiation of cellular SOD and glutathione peroxidase activities was noted [72]. Treatment of 3xTgAD mice with nicotinamide also resulted in improved cognitive performance, concordant with normalizing mitochondrial dynamics and increased expression of the fission protein, DLP1 in the cerebral cortex [68]. We have previously demonstrated that St. John's wort semisynthetic derivate THH counteracts oxidative insult in APP/PS1 mice brain, by reducing the formation of 4-HNE adducts and caspase-3 activation [36]. The underlying mechanism of action seems to be related to the prevention of mitochondrial Ca^2+^ overload, and modulation of the fusion-fission process, arresting mitochondrial dysfunction [36]. In the other hand, moderate exercise promotes increased activity of the mitochondrial complexes I, III and IV in the brain and prevents age-dependent mitochondrial decline reported in sedentary rodents [113].

As well, another study demonstrated that brain adaptations to endurance training included overexpression of PGC-1α and Sirt1 mRNA overexpression, together with increased mitochondrial DNA content, suggesting increased mitochondrial mass [114]. Similarly, it is also well known that exercise induces the regulation of brain mitochondrial redox balance, and chronic exercise reduces apoptotic signaling in the AD brain [115]. Finally, different approaches using 10 selected polyphenols, shown to ameliorate membrane disruption caused by the Aβ_42_ peptide, and tau-441 proteins, suggest that these abnormal protein aggregates might interfering with the mitochondrial membrane [116].

The maintenance of intracellular NAD^+^ levels in human brain cells may also be crucial for the retention of cellular viability during conditions of chronic oxidative stress and mitochondrial dysfunction through the promotion of oxidative phosphorylation (ATP production). NAD^+^ is also closely associated with the DNA binding family of enzymes known as poly (ADP-ribose) polymerases (PARPs) [117,118]. Under physiological conditions, PARP activation leads to DNA repair and recovery of normal cellular function. However, under pathological conditions, PARP activation leads to increased NAD^+^ turnover, leads to reduced ATP synthesis, and the cessation of all energy dependent functions and consequent cell death [119-121]. Increased levels of functional PARP enzyme have been reported in the frontal and temporal cortex more frequently than age-matched controls in postmortem brains of AD patients. Maintenance of intracellular NAD^+^ pools may reduce cellular injury. NAD^+^ treatment has been shown to reduce PARP-induced astrocyte death [122]. Additionally, NAD^+^ may also prevent neuronal injury by enhancing sirtuin activities and/or improving energy metabolism [123].

## Conclusion

As the world’s ageing population continues to increase and age appears to be a prominent risk factor for most neurodegenerative diseases, novel therapeutic regimens which delay the onset of age-related disorders are highly desirable. There are multiple connections between neurodegenerative diseases, such as increased oxidative stress, decreased autophagy, and formation of misfolded proteins, impaired neuronal metabolism and mitochondrial dysfunction. Central to the maintenance of cellular function, and particularly synaptic structure and function, and mitochondrial integrity are the proto-oncogene *Wnt*, AMPK, mTOR, Sirt1 and PGC-1α. *Wnt* signaling activation (Figure 2), which protects neurons against neurotoxic damage and, in this sense, represents a new perspective regarding the underlying pathobiology of AD. Furthermore, strong evidence suggests that AMPK might be key master controller of important metabolic pathways and is closely aligned with Sirt1 and mTOR activities. Moreover, the crosstalk between these main pathways, as well as, with secondary cellular mechanisms are far to be addressed, but the currently available evidence suggest a more than plausible connection between the pathways herein presented. Of course important questions should be answered in order to fulfill the inconsistencies of some observations. Mainly, that several research groups have conducted some clinical trials using different kinds of drugs, such as PPARγ agonists (TZD) or SIRT1 agonists (Res), with disappointing results [124]; however, according to our experience, these sort of negative results are not due to the lack of action of the drug, but probably because of the inner complexity of the disease, and the lack of understanding between live animal models and human physiological response [125-131]. Moreover, researchers still avoid considering the response of adjacent tissues as a result of systemic AD therapies; what if the systemic administration of some drug induces an alteration of the blood–brain barrier health, limiting the further benefits of the drug within the brain parenchyma? Today, AD research is usually focused to unveil limited areas of the disease with unsuccessful results when challenged in real patients, we believe that through renewed insight on the cellular and molecular mechanisms responsible for cellular and mitochondrial abnormalities reported in AD, efficient and safe translation of these signaling pathways into novel therapeutic alternatives against neuronal degeneration may shorten the gap between basic science and clinical research. The fast and efficient translation of innovative therapeutics into clinical candidates, and eventually approved therapeutics will improve outcomes for AD patients.

## Abbreviations

AMPK: 5' adenosine monophosphate-activated protein kinase; mTOR: Mammalian target of rapamycin; Sirt1: Silent mating-type information regulator 2 homolog 1; PGC1: Peroxisome proliferator-activated receptor gamma coactivator 1-alpha; AD: Alzheimer’s disease; Aβ: Amyloid beta; NFT: Neurofibrillary tangles; Fz: Frizzled; APP: Amyloid precursor protein; DSH: Disheveled; PKC: Protein kinase C; LRP6: Low density lipoprotein-related receptor protein; apoE4: Allele 4 of apo-lipoprotein E; PPAR: Peroxisome proliferator-activated receptor; Aβo: Aβ oligomers; AChE: Acetylcholinesterase; THH: Tetrahydrohyperforin; AChR: Acetylcholine receptors; C. elegans: *Caenorhabditis elegans*; STK11: Serine/threonine kinase 11; CaMKK: Ca^2+^/CaM-dependent protein kinase kinase β; PPase: Phosphatases; FOXO3: Forkhead box O3; LTP: Long-term potentiation; BACE1: β-secretase; BAG2: BCL2-associated athanogene 2; LAMP1: Lysosomal-associated membrane protein 1; GHB: γ-Hydroxybutyric acid; IDE: Insulin-degrading enzyme; TPM: Topiramate; LEV: Levetiracetam; NAD+: Nicotinamide adenine dinucleotide; GDH: Glutamate dehydrogenase; DLP1: Dynamin-like protein 1 protein; OPA1: Optic atrophy protein 1; NRF: Nuclear respiratory factor; 4-HNE: 4-hydroxynonenal; PHF: Paired helical filaments; TFAM: Mitochondrial transcription factor A; PARPs: Poly(ADP-ribose) polymerases; NO: Nitric oxide; SOD: Superoxide dismutase.

## Competing interests

The authors declare no conflict of interest related to this study.

## Authors’ contributions

JAG, JAR, JMZ, NB and NI drafted and wrote the manuscript. All authors read and approved the final manuscript.
